# Epidemiology, Pathophysiology, and Current Treatment Strategies in Stroke

**DOI:** 10.26502/fccm.92920399

**Published:** 2024-08-29

**Authors:** Zubair Ahmed, Fihr Chaudhary, Devendra K. Agrawal

**Affiliations:** Department of Translational Research, College of Osteopathic Medicine of the Pacific, Western University of Health Sciences, Pomona, CA 91766, USA

**Keywords:** Apoptosis, Cytotoxicity, Hemorrhagic stroke, Inflammation, Ischemic stroke, Mechanical thrombectomy, Neuroprotection, Oxidative Stress, Personalized medicine, Thrombolytic therapy

## Abstract

Both ischemic and hemorrhagic strokes are critical health issues and the incidence is on the rise. The rapid neurological degeneration that can occur with either type of stroke warrants prompt medical attention. In the article, we critically reviewed the literature examining their incidence, pathophysiology, and present treatment strategies. Clinical trials show conflicting findings, with ischemic strokes accounting for 87% of all strokes. Brain injury following an ischemic stroke results in cell death and necrosis, immune cells being the primary actors in the process of neuroinflammation. In order to develop neuroprotective drugs against ischemic stroke, detailed investigation of glutamate production and metabolism as well as downstream pathways controlled by glutamate receptors provides significant information on the underlying mechanisms. The permeability of the blood-brain barrier and the degradation of glutamine synthase are two potential mechanisms by which peritoneal dialysis accelerates brain-to-blood glutamate clearance and thus reduces glutamate levels in the brain after a stroke. Oxidative stress in an ischemic stroke disturbs the oxidant-antioxidant balance, which is particularly problematic for brain cells that are high in polyunsaturated fatty acids. Because of demographic factors like age, sex, race/ethnicity, and socioeconomic status, the incidence and prevalence of stroke differ across people and regions. For rapid diagnosis and treatment decisions, diagnostic imaging tools such as vascular imaging, CT, and MRI are essential. To aid in the recovery and lessen neurological impairments following a stroke, novel avenues of research are under investigation on neuroprotective medications that target inflammation, oxidative stress, and neuronal death.

## 1. Introduction

Stroke has been typically described as a neurological deficit that is caused by an acute focal injury to the central nervous system due to vascular etiologies. However, this classical definition is mainly a clinical description and does not take into account the recent scientific and technological advancements that have been made regarding stroke outcomes. In 2013, the American Heart Association proposed an updated definition of stroke for the 21st century that integrates both clinical and tissue criteria. The definition accounts for permanent damage to the brain, spinal cord, or retinal cell death due to vascular dysfunction based on pathological or imaging evidence, in the presence or absence of symptoms [1]. Generally, there are three main classifications of stroke: ischemic, hemorrhagic, or transient ischemic attack (TIA). Ischemic stroke is defined as disrupted blood flow to a region of the brain due to obstruction of the blood vessels that supply it, leading to inadequate oxygen delivery, ischemia, and neuronal death [2]. Hemorrhagic stroke results from the rupture of blood arteries resulting in internal bleeding in the brain leading to a devastating effect on the parenchyma of the brain, damaging brain cells and may also lead to increased intracranial pressure and spasm in the blood vessels [3]. A TIA is a temporary blockage of blood flow to the brain, usually due to a blood clot that resolves on its own, with symptoms lasting less than five minutes [3]. Ischemic stroke accounts for 87% of all stroke cases and hemorrhagic stroke accounts for about 13% [4].

An INTERSTROKE study is an international case-control study where the cases are patients who have a first-time stroke and imaging (CT or MRI) is conducted within the first 72 hours of presenting to the hospital [5]. The first INTERSTROKE study investigated the proportions of ischemic and hemorrhagic stroke in 22 African countries. It was found that 66% of patients had ischemic stroke and 34% of patients had hemorrhagic stroke [6]. These proportions of the different subtypes of stroke were later confirmed by another study, the Stroke Investigative Research and Educational Network (SIREN). They analyzed the patient cases from Nigeria and Ghana and found that 68% of patients were diagnosed with ischemic stroke and 32% had hemorrhagic stroke [7].

Ischemic stroke can be further classified into several other subtypes based on the classification system that is being used. There are numerous classification systems such as Causative Classification System (CCS), Atherosclerosis, Small‐Vessel Disease, Cardiac Source, Other Cause (ASCO), and Chinese Ischemic Stroke Subclassification (CISS). At this time, the most widely used international classification system is the Trial of ORG 10172 in acute stroke treatment (TOAST) classification scheme [8]. TOAST categorizes ischemic stroke into five main etiological subtypes: large artery atherosclerosis, small artery occlusion, cardio embolism, stroke of other determined causes, and stroke of undetermined cause [8]. Although TOAST is the most commonly accepted system and relatively simple to utilize, there are some drawbacks. For example, the accuracy and reliability of diagnosing small vessel disease-related infarcts is significantly reduced because of the 15 mm lesion cut-off point. For CT imaging studies, this measurement was typically used as the maximum size limit for small vessel infarcts, but MRI imaging has shown these lesions can increase up to 20 mm. Thus, these size restrictions are not accurate [9]. Another disadvantage is that TOAST work-up usually stops when one etiology is identified which can lead to inaccurate diagnoses and treatment plans for patients. Thus, modified TOAST classifications were implemented, such as SSS-TOAST, to correct these issues and improve accuracy [9].

Furthermore, hemorrhagic stroke can also be subclassified into two major types: intracerebral hemorrhage and subarachnoid hemorrhage. Intracerebral hemorrhage is the most common type as it accounts for 80% of all hemorrhagic strokes [10]. It is defined as bleeding into the brain parenchyma and is most commonly caused by uncontrolled hypertension in small penetrating arteries such as the anterior, middle, and posterior cerebral arteries [11]. Subarachnoid hemorrhage is the accumulation of blood in the subarachnoid space, the area between the pia mater and the arachnoid membrane [12]. It is most commonly caused by saccular aneurysms, but it is also associated with intracranial neoplasms, arteriovenous malformations, and some anticoagulants.

## 2. Prevalence and Incidence

Globally, stroke is the second leading cause of death and a significant contributor to long-term disability [13]. According to the Global Burden of Disease Study 2020, the global prevalence of all stroke subtypes of 89.13 million cases and the age-standardized stroke prevalence rates were highest in sub-Saharan Africa and in certain regions of the Southeast United States and East and Southeast Asia. More specifically, the global prevalence of ischemic stroke was 68.16 million cases and the age-standardized stroke prevalence rates were highest in sub-Saharan Africa and in the Eastern United States. The global prevalence of intracerebral hemorrhage was 18.88 million cases with the age-standardized stroke prevalence rates being highest in Southeast Asia, western sub-Saharan Africa, and Oceania. Subarachnoid hemorrhage has a global prevalence of 8.09 million cases and the age-standardized stroke prevalence rates were highest in Latin America and Japan [14]. Regarding incidence, the global incidence of stroke was 11.71 million people, and the incidence of ischemic stroke, intracerebral hemorrhage, and subarachnoid hemorrhage was 7.59 million, 3.41 million, and 0.71 million people, respectively [14]. Globally, in terms of mortality, of the 7.08 million people who died from stroke in 2020, 49% of total deaths were due to ischemic stroke. Intracerebral hemorrhage and subarachnoid hemorrhage were the cause of 46% and 5% of total stroke-related deaths, respectively [14]. In the United States alone in 2021, 1 in 6 cardiovascular disease-related deaths were due to stroke. Furthermore, someone has a stroke every 40 seconds and someone dies from a stroke every 30 minutes and 14 seconds [15]. Undoubtedly, on a local and international level, stroke has placed a significant burden on the lives of stroke patients and the surrounding community.

Transient ischemic attack (TIA) occurs due to a transient obstruction of blood flow to the brain that results in temporary neurological deficits that typically resolve on their own. Although TIAs are relatively common, affecting about 240,00 people in the United States every year, it can be difficult to determine the incidence and prevalence since the symptoms that are typically experienced may not be neurologically specific and they only last for a brief moment in time [16]. Thus, many patients may not seek medical care and if they choose to do so, by the time they interact with a medical professional, their symptoms have resolved. Therefore, the current data regarding TIA epidemiology is quite variable, but studies have shown that the prevalence of TIAs within the United States is approximately 2.3% [17]. Additionally, a study from 2014 investigated the incidence rates of TIAs in Europe. It was found that the incidence rates were 0.52–2.37 in men and 0.05–1.14 in women aged 55–64, 0.94–3.39 in men and 0.71–1.47 in women aged 55–74, 3.04–7.20 in men and 2.18–6.06 in women aged 75–84[18]. The incidence rates increased with age and were higher in men compared to women. Moreover, the United States had similar TIA incidence rates but it was low in Japan [18].

## 3. Risk Factors of Stroke

### a. Modifiable Risk Factors

Due to the relatively high incidence and prevalence of stroke across the globe, stroke prevention is one of the most heavily investigated areas of interest in order to reduce the burden of stroke on the community [19]. Through comprehensive epidemiological and longitudinal studies, multiple risk factors have been identified that play an instrumental role in primary and secondary stroke prevention. The risk factors can typically be categorized into two separate groups: modifiable and non-modifiable risk factors. Modifiable risk factors are susceptible to various interventions to help reduce the risk of stroke whereas non-modifiable factors cannot be controlled and serve as indicators for high risk of stroke. A recent INTERSTROKE study from 2016 analyzed the potential effects of modifiable risk factors associated with stroke in 32 countries. Among the 13,000 stroke cases, it was shown that modifiable risk factors such as diet, physical inactivity, hypertension, psychosocial factors, cardiac causes, diabetes, smoking, abdominal obesity, hyperlipidemia, and alcohol consumption accounted for approximately 90% of all strokes [20]. Furthermore, the Global Burden of Disease study highlighted that 90.5% of all strokes can be explained by modifiable risk factors as well [21,22].

Hypertension is the most important modifiable risk factor for stroke. A recent study from 2014 estimated that over 103 million adults in the United have been diagnosed with hypertension and patients with hypertension are three or four times more likely to have a stroke [23,24]. The Framingham Heart Study is an ongoing longitudinal epidemiologic study that has been going on since 1948 to investigate different risk factors for stroke and other cardiovascular diseases [25]. After analyzing results from the first cohort of subjects, it was found that hypertensive patients with a blood pressure (BP) >160/95 mmHg had a five to 30 times higher likelihood of having a stroke compared to normotensive patients who had a BP <140/90 mmHg [26]. These results led to the creation of the Framingham stroke prediction algorithm which now takes into account age, smoking, history of cardiovascular diseases, and other risk factors. Additionally, the Prospective Study Collaboration is a combination of 61 prospective studies that explored established risk factors’ effects on mortality rates from different vascular causes such as stroke [27]. Results from this study concluded that patients between the ages of 40–89 had a strong correlation between blood pressure and total vascular and stroke mortality. Moreover, the risk of death from stroke doubles with every 20 mmHg increase in systolic blood pressure or 10 mmHg increase in diastolic blood pressure. This association remained consistent down to a blood pressure of at least 115/75 mmHg [28]. Additionally, average blood pressure was a more accurate predictor of stroke-related deaths than either systolic or diastolic blood pressure measurements. Systolic blood pressure provided more information regarding stroke mortality than either diastolic blood pressure or pulse pressure. These trends were found to be similar in both male and female patients as well [29].

Diabetes is another well-established modifiable risk factor for stroke. Approximately 537 million people have diabetes worldwide and this number is projected is to increase to 643 million by 2030 and 783 million by 2045 [30]. In the Emerging Risk Factors Collaboration study, the hazard ratios with diabetes for ischemic stroke, hemorrhagic stroke, and unclassified stroke were 2.27, 1.56, and 1.84, respectively [31]. Furthermore, in a Greater Cincinnati/Northern Kentucky stroke study, it was found that younger individuals with diabetes had a higher risk of stroke [32]. The incidence of stroke increased in all age groups, the risk of stroke was much more prominent before the age of 65 in Whites and before the age of 55 in African American populations [33].

### b. Non-modifiable Risk Factors

There are relatively few non-modifiable risk factors, and the most significant ones are age and sex. Age is the strongest non-modifiable risk factor for stroke and elderly patients with strokes typically have higher mortality rates and reduced ability for functional recovery [34]. Thus, as you age, there is a higher chance of developing a stroke. The likelihood of having a stroke double after every 10 years after the age of 55 [35]. However, even though stroke is more prevalent in older populations, about one in seven strokes take place in people between the ages of 15 and 49 due to the presence of modifiable risk factors such as high blood pressure, diabetes, and obesity [36]. Another study investigated stroke outcomes and its subtypes in younger populations compared to older populations and found that patients younger than the age of 45 had a higher incidence of hemorrhagic stroke compared to other age groups. The most significant risk factor associated with this trend was hypertension in young adults [37]. Furthermore, case fatality and in-hospital mortality rates due to ischemic stroke increase with age [38]. Patients with ischemic stroke, greater than the age of 80, were less likely to be discharged to their pre-stroke residence and had a longer length of stay at the hospital compared to younger individuals [39]. Their case fatality at discharge can increase to as high as 24.2% [39].

Sex differences play an integral in stroke outcomes as well. Historically, it has been well-documented that the incidence of stroke is higher in men compared to women [40,41]. However, recent studies have shown that there is a decrease in ischemic stroke among men which means that the incidence of stroke is declining more in men than women and the overall incidence of stroke is decreasing over time [42]. Furthermore, women also tend to have increased disability post-stroke, decreased quality of life, and overall worse outcomes likely due to depression, anxiety, pain, and decreased mobility compared to men [43].

## 4. Underlying Conditons and Pathophysiology of Stroke

### a. Hypertension

As referenced before, hypertension is a prominent determinant of stroke mortality due to its significant effect on cerebral circulation [44]. During periods of hypertension, the high levels of pressure within the lumen of blood vessels can lead to endothelial dysfunction and alter the function of smooth muscle lining the walls of these vessels. This decreases the size of the diameter of the lumen and will reduce the ability of cells to release vasodilatory factors such as nitric oxide. This lack of vasodilation causes an increase in vasoconstrictor tone of both systemic and cerebral arteries [10,44]. Furthermore, damage to the endothelium can lead to ischemic lesions and thrombus formation. Additionally, hypertension can lead to atherosclerosis and degenerative changes to the endothelium and smooth muscle, ultimately leading to the development of intracerebral hemorrhages [45].

### b. Diabetes

There are numerous pathophysiological mechanisms regarding how diabetes can lead to stroke. The most common mechanisms include endothelial dysfunction, arterial stiffness, systemic inflammation, and thickening of the capillary basal membrane [33]. Left ventricular diastolic filling is a common problem seen in patients with type II diabetes. The proposed mechanism behind the development of congestive heart failure in these patients, which can lead to stroke, is microvascular disease, autonomic dysfunction, interstitial fibrosis, and most notably, hypertension [33]. As noted previously, hypertension causes decreased availability of nitric oxide, a potent vasodilator, which leads to endothelial dysfunction and atherosclerosis [46]. Moreover, the formation of atherosclerotic plaques is exacerbated by an enhanced inflammatory response, which is commonly seen in diabetic patients.

## 5. Pathophysiology of Ischemic Stroke vs Hemorrhagic Stroke

Clinical trials comparing ischemic (IS) and hemorrhagic (HS) strokes have shown contradictory findings. Disabilities and fatalities caused by stroke rank among the highest in the globe. Acute treatments have made ischemic stroke, which accounts for 87% of all strokes, a time-dependent condition [47]. Two types of hemorrhagic strokes are intracerebral hemorrhage (3% of all strokes) and aneurysmal subarachnoid hemorrhage (10% of all strokes) [47]. The leading causes of death in 2017 were ischemic stroke (2.7 million), intracerebral hemorrhage (3 million), and aneurysmal subarachnoid hemorrhage (0.4 million) [47]. The overall outlook for patients suffering from ischemic stroke is deemed more favourable compared to hemorrhagic stroke, which is characterized by a higher mortality rate, particularly in the immediate and subsequent stages [48,49].

### a. Ischemic stroke

Ischemic strokes happen when blood flow to a portion of the brain is impaired due to a blocked or “clogged” blood vessel. Within minutes, the brain cells and tissues start to die due to a shortage of oxygen and nutrients. A thrombus, or blood clot, forms in the arteries that carry blood to the brain, leading to a stroke known as a thrombotic stroke [50, 51]. Post-operative atrial fibrillation and high risk anesthesia procedures are risk factors for the development of stroke [52,53]. Stroke of this kind most commonly affects the elderly, and it is more common in those who have diabetes, high cholesterol, or atherosclerosis (the hardening of the artery walls due to the accumulation of fat and lipids). The atherosclerotic plaques become unstable due to increased inflammation, apoptosis of carotid artery smooth muscle cells, and increased matrix metalloproteinases that degrade extracellular matrix [54–56]. Many inflammatory mediators and cytokines are involved in plaque instability leading to the migration of plaque particles and thrombus to brain arteries and arterioles resulting in the blockade of blood flow to brain cells leading to ischemia and stroke symptoms [57–60]. This Sudden onset of symptoms, particularly in the middle of the night or first thing in the morning, is common in cases of thrombotic stroke [50]. Sometimes it happens suddenly, and other times it could take hours or days. Preceding thrombotic strokes are “mini strokes,” sometimes known as transient ischemic attacks (TIAs). TIAs are a common precursor to stroke and can be short- or long-lived, lasting anything from a few minutes to a whole day. Although TIA symptoms are often minor and temporary, they are comparable to stroke symptoms. An further kind of stroke known as a lacunar infarct can happen in the brain’s tiny blood arteries. Originating in Latin, the term “lacunar” means “hole” or “cavity.” Diabetes and hypertension are common causes of lacunar infarctions. A typical culprit in embolic strokes is a blood clot, or embolus, which develops in another part of the body and makes its way to the brain via the circulatory system. Strokes caused by embolism can happen suddenly and with no warning symptoms; they are common complications of heart disease or cardiac surgery. Individuals experiencing atrial fibrillation, a cardiac rhythm abnormality characterized by inefficient pumping of the upper chambers, account for approximately 15% of all embolic stroke [52,61].

### b. Hemorrhagic stroke

In a hemorrhagic stroke, a blood artery that delivers blood to the brain bursts and bleeds. There is a lack of oxygen and nutrients for brain cells and tissues when an artery bleeds into the brain. Pressure also builds up in the surrounding tissues, causing inflammation and swelling—all of which can worsen the brain damage already there. Excessive blood pressure is the most common cause of intracerebral hemorrhage. Instantaneous and fast bleeding ensues. Coma or death may result from bleeding that has no apparent cause. When the brain bleeds into the subarachnoid space, it creates a meningeal hematoma. The most common causes of this kind of bleeding are arteriovenous malformations (AVMs) and aneurysms. Also, traumatic events can trigger these conditions [62].

## 6. Pathophysiology of Ischemic Stroke

The intricate pathology of an ischemic stroke involves a cascade of events that culminate in neuronal death and necrosis in the affected region, including oxidative stress, inflammation, excitotoxicity, and the complement system. Inflammation plays an increasingly important role in the complicated pathophysiology of Ischemic Stroke; this function was previously underappreciated due to the brain’s long-held reputation as an immune-privileged organ. Research on excitotoxicity, which changed the face of stroke in the 1980s [63], is primarily caused by elevated glutamate levels and the resulting calcium excess. Damaged ischemic tissue, especially following reperfusion, is especially vulnerable to oxidative stress. Important mechanisms that promote ischemic stroke and consequent neuronal death include activation of the complement system, development of thrombus, and pericyte death.

### Inflammation

**Figure 1:**
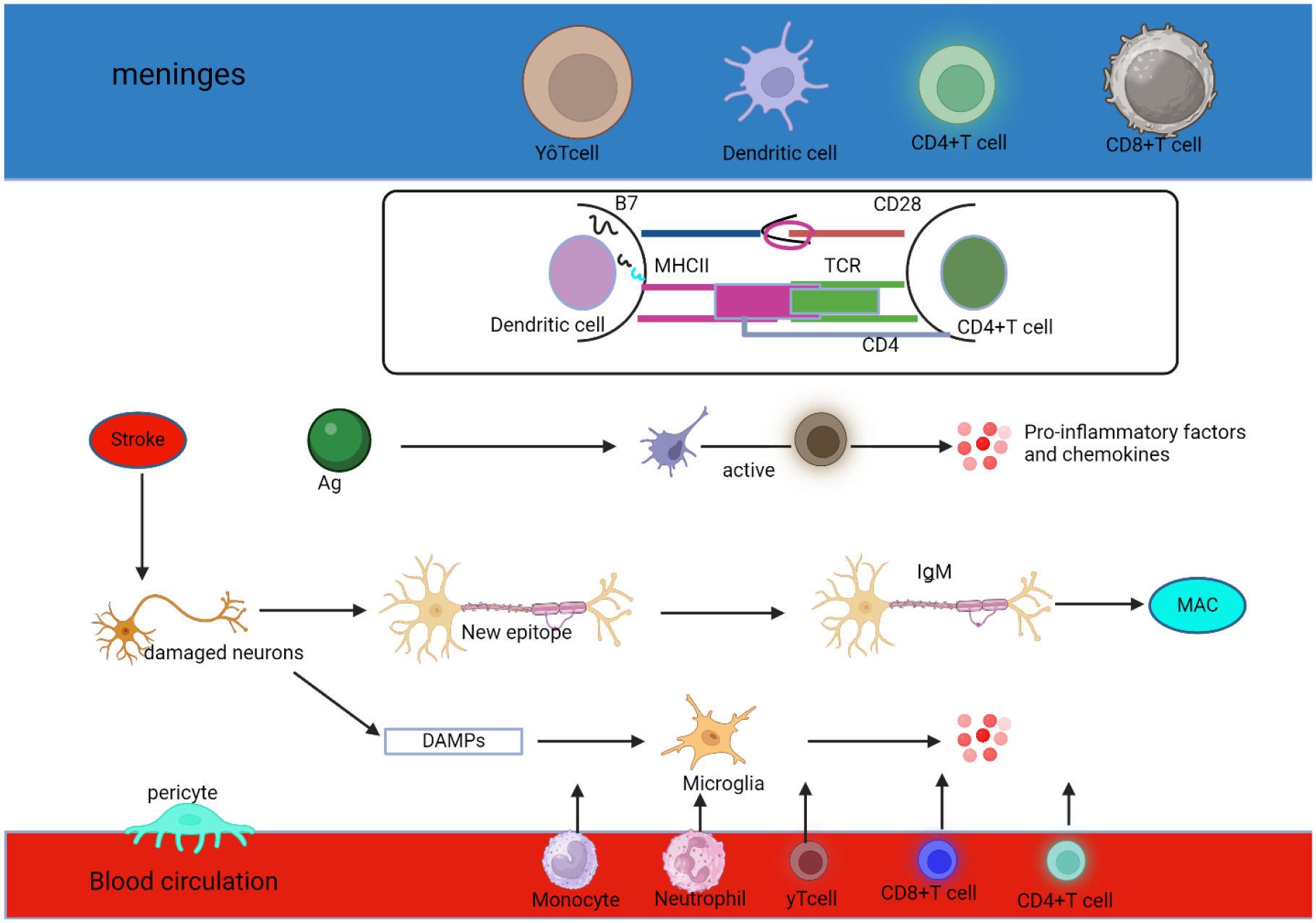
Inflammation in the ischemic stroke. For example, MHC II-peptide-TCR and CD28-B7 activate CD4+ T cells, which in turn increase T cell activation. Dendritic cells (DCs) consume, and process antigen (Ag) produced by injured neurons. While responding to an inflammatory stimulus, activated T cells release chemokines and other substances that promote inflammation. Natural immunoglobulin IgM in brain tissue can recognize novel epitopes exposed to neurons after necrosis, which activates the classical pathway of the complement system and finally forms a membrane attack complex (MAC). Later on, MAC releases intracellular substances, which makes the inflammatory reaction worse. Thirdly, as part of the inflammatory response, damaged neurons secrete DAMPs, which activate microglia. 4. Inflammatory cells like microglia release chemokines that encourage peripheral phagocytes, neutrophils, CD4+T cells, CD8+T cells, and γδT cells to go towards the central nervous system.

Ischemic stroke is accompanied by neuroinflammation, which is primarily caused by immune cells (both innate and adaptive). Damage to the brain after an ischemic stroke causes cell death and necrosis, which triggers an inflammatory response regulated by the release of cytokines, chemokines, and reactive oxygen species (ROS) (Figure 1). This process initiates the microcirculation and involves many cytotypes, including microglia and lymphocytes, which lead to neuronal death [64]. Injuries to the nervous system can manifest in different ways depending on their location, timing, and severity. In the immediate aftermath of a stroke, microglia are involved in neuroinflammation in two ways. Through their targeting of microglia, microRNAs such as miR-203 have been discovered to reduce cerebral ischemia-reperfusion injury [65]. In addition, microglial polarization, and especially M1 polarization, has been linked to worsening cerebral ischemia [66]. Acute stroke is characterized by severe neuroinflammation, which is associated with neuronal damage, a disruption of the blood-brain barrier (BBB), and poor prognoses [67]. Ischemic stroke is a leading cause of death and disability, and the effectiveness of treatment depends on how well it prevents neuronal death.

### Excitatory toxicity

An increase in glutamate (Glu) levels in an ischemic brain triggers a cascade of metabolic reactions that ultimately cause neuronal death and toxic excitability. The reduction in mitochondrial ATP generation following an acute stroke is a result of cerebral ischemia and hypoxia [68]. Glutamate levels in the extracellular or synaptic cleft will rise as a result of intracellular and extracellular ion disorders induced by a defective ATP-dependent ion pump. Intracellular ion disorders include an increase in Na^+^, Ca^2+^, and Cl^−^, while extracellular ion disorders have an increase in K^+^. One example is the ATP-dependent Na+ pump, which controls the intracellular transport of glutamate, which is reliant on a normal Na^+^ gradient (extracellular Na+ are greater than intracellular). When ATP synthesis is inhibited and the ATP-dependent Na+ pump is deactivated, the typical Na+ gradient is inverted, meaning that internal Na^+^ are higher than external Na^+^. This leads to an increase in glutamate in the extracellular matrix or synaptic cleft. Researching glutamate synthesis and metabolism as well as the downstream pathways regulated by glutamate receptors has enormous promise for the creation of neuroprotective medications against ischemic stroke, since glutamate-mediated excitotoxicity greatly impacts stroke prognosis [68].

Scientists have shown that peritoneal dialysis can lower brain glutamate levels following a stroke [69]. Some possible primary causes of this occurrence are as follows. Breakdown of the blood-brain barrier came first. Following a stroke, there will be a gradient between the levels of glutamate in the brain and the blood, with the former having greater concentrations [70,71]. It is possible that glutamate can enter the periphery if the BBB is disrupted. Furthermore, glutamine is a neurotransmitter that can directly pass the blood-brain barrier and enter the periphery. Peritoneal dialysis flushes out the glutamine-regenerated peripheral glutamate. Nerve cells and the astrocytes that surround them participate in a glutamate-glutamine cycle [72,73], and extracellular glutamate synthesis relies on several targets in this cycle. Members of the family of transporters known as excitatory amino acid transporters (EAATs) include glutamate transporter GLT-1, which can empty the synaptic cleft of glutamate. According to reference [74], β-lactam antibiotics have the ability to enhance GLT-1 activity in glial cells of mice following OGD. Reactive oxygen species (ROS) can destroy glutamine synthase (GS), which is the speed limit of the glutamate-glutamine cycle. This degradation can occur after a stroke, leading to a buildup of glutamate [75]. One potential solution to the problem of ischemic stroke is to target GS.

### Oxidative stress

A key component of ischemic stroke, oxidative stress upsets the oxidant-antioxidant equilibrium, especially in brain cells that are rich in polyunsaturated fatty acids. A number of factors exacerbate oxidative damage, including an increase in oxidative metabolism, a lack of antioxidants, and high levels of pro-oxidants (such as iron) [76].

Calcium homeostasis is upset during an acute ischemic stroke, which leads to a release of calcium into the brain and the activation of pathways that generate reactive oxygen species (ROS) and oxidative damage. The brain is tremendously damaged by the overabundance of reactive oxygen species (ROS) and hydroxyl radicals caused by these oxidants and antioxidants imbalance [77]. Ischemic stroke leads to an increase in cellular ROS production as a result of glucose and oxygen deprivation, which worsens oxidative stress and brain damage [78]. The main enzymes responsible for producing superoxide anion during ischemia are xanthine oxidase (XO) and NADPH oxidase (NOX). A buildup of hypoxanthine and xanthine, which are XO substrates, induces ATP depletion during ischemia and the subsequent formation of reactive oxygen species (ROS) [79,80]. Following an ischemic stroke, there is an increase in XO expression in the area just outside of the infarcted tissue. NOX is an additional important source of reactive oxygen species (ROS), and its upregulation follows a stroke. Specifically, NOX2 is the principal NOX that produces superoxide when triggered by the N-methyl-D-aspartate receptor [81].

In ischemic neuronal death, mitochondria, the “cellular powerhouses,” play an essential role in regulating the amount of energy a cell uses. Damage to mitochondrial respiratory activity and membrane potential sets off a chain reaction that ultimately causes neuronal death during ischemia. Excessive reactive oxygen species (ROS) production, decreased ATP generation, increase of PTEN-induced putative kinase 1 (PINK1) and unfolded protein response (UPR) are all symptoms of mitochondrial depolarization [82]. Increases in reactive oxygen species (ROS) and calcium levels trigger the opening of the membrane permeability transition pore (MPTP), which in turn releases cytochrome c. This activation sets off effector caspases, which carry out apoptotic death [83]. As soon as PINK1 detects mitochondrial damage, it initiates the damage process by recruiting Parkin and phosphorylating Parkin and ubiquitin [84].

### Apoptosis

In apoptosis, neurons undergo a shrinkage and cytoplasmic condensation process that culminates in the nuclear membrane being ruptured, resulting in the formation of apoptotic bodies. These events can be either intrinsic or extrinsic. When the oxygen and nutrition levels of the cells drop too low, the intrinsic system kicks in and stops the normally occurring glycolytic oxidative phosphorylation pathway from producing ATP. Because of this, the anaerobic pathway is most common, and the amount of ATP that cells make is inadequate to sustain their activity. An imbalance in the ion channels (Na^+^/Ca^2+^ influx and K^+^ efflux) and an accumulation of calcium ions within the cell lead to an overproduction of glutamate and other excitatory amino acid neurotransmitters that are released into the extracellular environment. A series of cytotoxic events in the nucleus and cytoplasm ensue after this process, including calpain activation, ROS generation from mitochondrial metabolism, DNA damage, and DNA breakage [85]. The extrinsic pathway, which can happen alone or in combination with the intrinsic pathway, includes the activity of inflammatory signaling factors released by astrocytes, microglia, and oligodendrocytes as a result of cerebrovascular damage. Proinflammatory cytokines and receptors such as TNF-α/β, chemokines, interleukin 1β, TNF-related apoptosis-inducing ligand receptor (TRAIL-R), and Fas ligand (FasL) are components of these causes of inflammation [86]. Neuronal cell membrane receptors mediate apoptosis via the mitochondrial-dependent pathway, which is initiated by a signal produced by caspase-8 and activates the downstream effector caspase-3 or BID [87].

**Figure 2:**
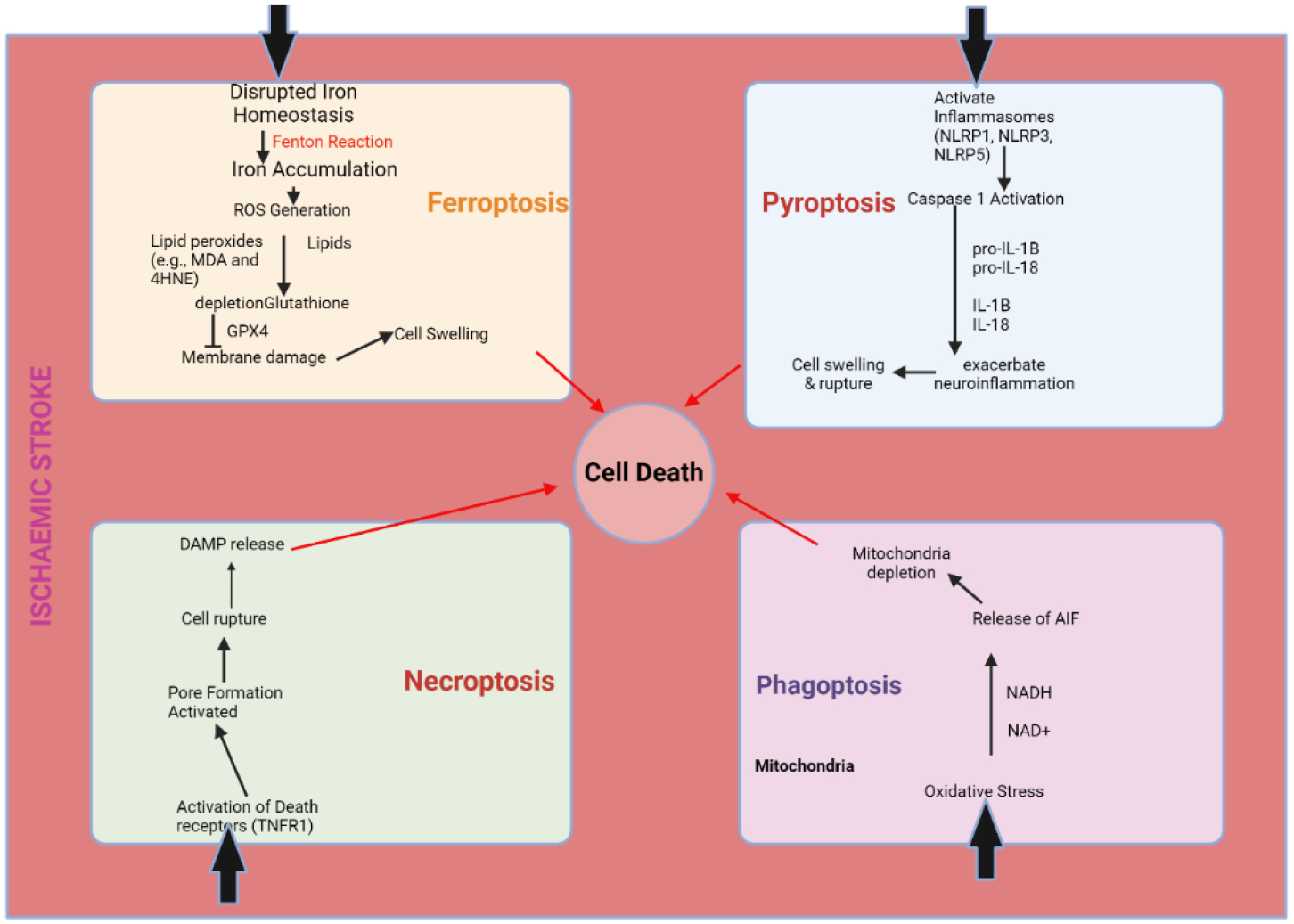
Different Forms of Cell Death Associated with Ischemic Stroke.

Ferroptosis, phagoptosis, parthanatos, pyroptosis, and necroptosis are five other ways in which cells might die after an ischemic stroke (Figure 2), in addition to apoptosis [88]. If we want to create effective treatments for ischemic stroke, we need a better understanding of how these many cell death mechanisms interact with one another. Improving outcomes for individuals suffering from ischemic stroke and reducing neuronal damage should be possible with the use of combined knowledge from these pathways.

## 7. Hemorrhagic Stroke Pathology

The fast buildup of blood within the brain parenchyma causes abnormalities in normal anatomy and elevated local pressure during intracerebral hemorrhage. Most of the harm, depending on the growth dynamics of the hematoma, happens mechanically due to the mass effect and happens between minutes to hours after bleeding begins. In most cases, the presence of intraparenchymal blood is to blame for secondary injury, the severity of which can vary with factors like the initial hematoma volume, age, and ventricular capacity [89]. Numerous concurrent pathogenic mechanisms might lead to its occurrence, such as: (1) blood cytotoxicity [90]; (2) hypermetabolism [91]; (3) excitotoxicity [92]; (4) spreading depression [93]; and (5) oxidative stress and inflammation [94]. The pathology culminates in the irreparable breakdown of the neurovascular unit, which includes both white and grey matter, as well as the blood-brain barrier, fatal brain edema, and the death of a large number of brain cells [95]. On one hand, inflammatory mediators produced locally in reaction to brain death or injury can amplify damage from ICH (secondary injury). On the other hand, inflammatory cells like microglia and macrophages are essential for clearing out cellular debris from hematomas, which is where inflammation continues [96].

### Blood Cytotoxicity and Oxidative Stress as Mediators of Cell Death After Hemorrhagic Stroke

The common sites of the bleed are the basal ganglia (50%), cerebral lobes (10% to 20%), the thalamus (15%), pons and the brain stem (10% to 20%), and the cerebellum (10%) [97]. Hemorrhage damages neurons and glia. Swelling of cells, malfunction of the mitochondria, neurotransmitter release, and oligemia are the outcomes. Inflammation and edoema are brought about by thrombin’s activation of microglia [98, 99]. Hemorrhage and subsequent rise in intracranial pressure (ICP) compress brain tissue, causing the main harm [100]. The release of hemoglobin and iron from the clot, as well as inflammation, BBB disruption, edoema, reactive oxygen species (ROS) overproduction, and glutamate-induced excitotoxicity, all play a role in secondary damage.

In most cases, the hematoma will swell within three to twelve hours. In one-third of cases, the hematoma enlarges within three hours. A 24-hour rise, a peak around 5–6 days, and a duration of up to 14 days characterise the perihematomal edoema. The area surrounding the hematoma is not receiving enough blood flow. The progression of ICH can be triggered by events such as a hematoma’s enlargement, intraventricular hemorrhage, swelling around the hematoma, and inflammation [97]. By first compressing the fourth ventricle, cerebellar hematoma causes hydrocephalus.

Perimesencephalic and non-perimesencephalic subarachnoid hemorrhages are two types of non-aneurysmal spontaneous subarachnoid hemorrhages. The interpeduncular cistern is the most common site of bleeding in perimesencephalic SAH. An increased risk of perimesencephalic nonaneurysmal SAH (PM-SAH) can be observed in individuals who have experienced physical activity, such as the Valsalva manoeuvre, which leads to an increase in intrathoracic pressure and raised intracranial venous pressure [101]. In non-perimesencephalic Sudden Anaphylaxis (NPM-SAH), the blood is distributed diffusely [102].

**Figure 3:**
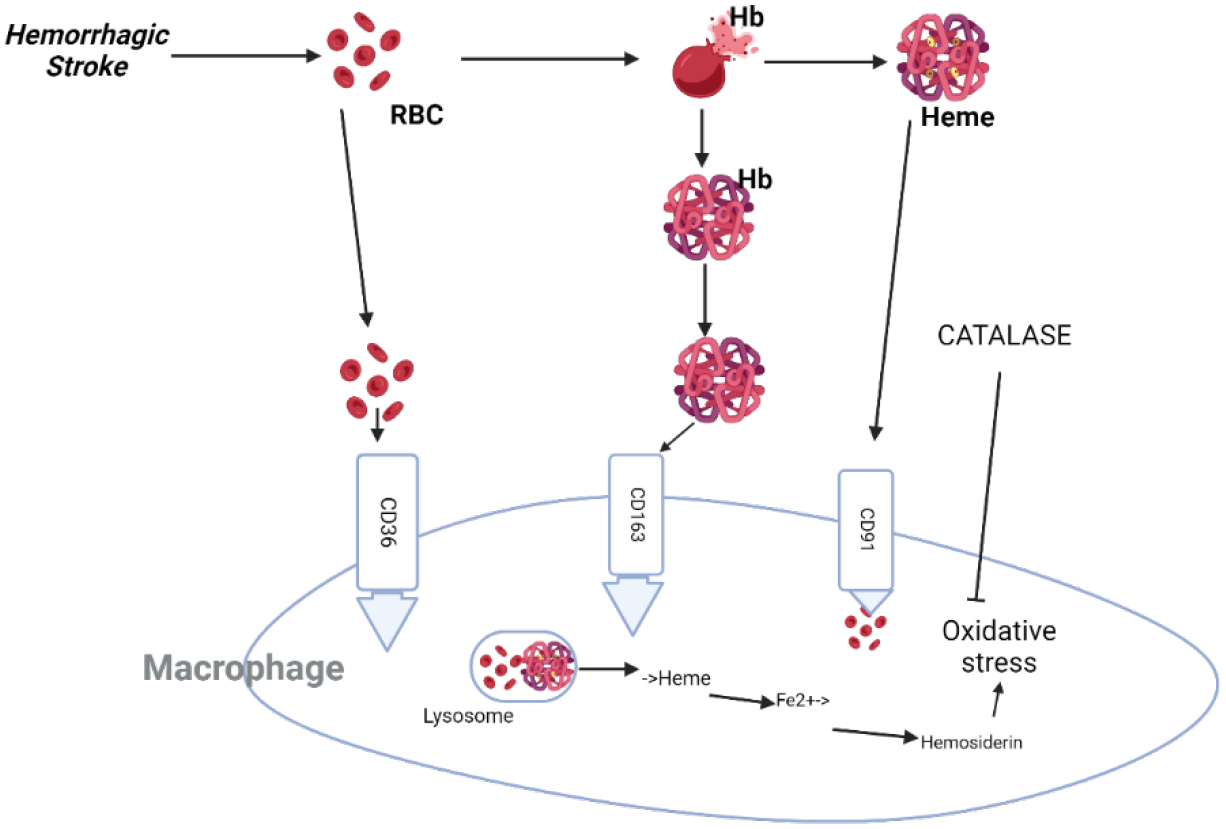
The process of intracerebral hemorrhage (ICH) involves the leakage of blood into the brain tissue. Blood cells, or erythrocytes, are removed from the parenchyma by macrophages and microglia via phagocytosis mediated by the cell-surface scavenger receptor CD36. Efficient removal of extravasated red blood cells and irreparably damaged cells stops them from lysing and releasing their harmful contents into the brain parenchyma. For hemolysis to take place (which happens after ICH), hemoglobin (Hb) must be eliminated from the extracellular space as soon as possible to prevent its cytotoxic effects. The protein haptoglobin (Hp) is brought into the brain from the blood and made locally by oligodendroglia. It forms a less hazardous compound with hemoglobin (Hb), which microglia and macrophages endocytose through the scavenger receptor CD163. When hemoglobin binds to hemopexin (Hx), it neutralises the harmful extracellular free heme that is produced. Phagocytes then remove the heme-Hx complexes through endocytosis mediated by the CD91 scavenger receptor. In phagocytes, biliverdin, CO, and pro-oxidative iron are produced through heme oxygenase (HO; mostly, HO-1) metabolism. To protect cells from oxidative stress, phagocytes use iron-binding proteins like ferritin or hemosiderin to store iron. When the iron-storage capacity of hemosiderin is reached, free irons create oxidative damage. This can happen when iron production is excessive. Although PPARγ regulates the expression of CD36, Nrf2 increases the expression of Hp. Nrf2 and PPARγ both promote the upregulation of antioxidant proteins, including as catalase and superoxide dismutase.

Within minutes of intracranial hemorrhage (ICH), viable brain cells nearby are subjected to a powerful cytotoxic, pro-oxidative, and proinflammatory insult from the blood’s extravasated components (mainly erythrocytes and plasma proteins) as well as damage-associated molecular patterns (such as nucleic acids, extracellular matrix components, proteins, lipid mediators, ATP, and uric acid released from necrotic and damaged tissue) (Figure 3). It is currently believed that bioactive molecules such as immunoglobulins, blood-derived coagulation factors, complement components, and extravasated plasma components contribute to tissue damage caused by ICH [103]. As a result, the lysis of red blood cells begins at approximately 24 hours and persists for the following several days, resulting in the release of cytotoxic hemoglobin (Hb) and further worsening of the clinical condition [104]. Hemoglobin and its byproducts, iron and heme, pose a direct threat to the health of nearby brain cells [105]. Hemoglobin and heme are powerful cytotoxic agents that can kill a large number of brain cells (Figure 3). Hb toxicity mostly manifests as free radical generation and extensive oxidative damage to proteins, nucleic acids, carbohydrates, and lipids, primarily by a Fenton-type mechanism [106]. The clinical significance of determining how to regulate hemolysis, heme detoxification, and iron intake could thus be substantial.

### Oxidative Stress and Hemorrhagic Stroke Injury

It seems that oxidative stress is a major factor in the development of ICH. By showing that antioxidants were effective as treatment agents, we were able to provide direct evidence that free radicals caused ICH injuries. In animal models of ICH, brain injury was greatly decreased by free radical scavengers like dimethyl thiourea, α-phenyl-N-tert-butyl nitrone, NXY-059 (a sulfonyl derivative of α-phenyl-N-tert-butyl nitrone), or deferoxamine, a medication that chelates pro-oxidative iron [107]. Consistent with these pharmacological studies, post-ICH damage was less in animals lacking NADPH oxidase, an enzyme critical for ROS generation [108]. New evidence suggests that estrogen mitigates ferrous iron toxicity both in living organisms and in laboratory settings, suggesting that variations in iron toxicity management could contribute to gender differences in ICH susceptibility [109].

Clinical trial results with NXY-059 were unexpectedly unsatisfactory, despite the existence of preclinical evidence for the efficacy of free radical neutralization-based therapies. Not only did NXY-059 not help individuals with ischemic stroke, it was also unsuccessful in treating ICH [110]. Inadequate stoichiometric neutralization of high amounts of free radicals and the drug’s pharmacokinetics (no blood-brain barrier permeability) are two potential causes of these neutral outcomes; however the exact explanation is unknown.

### Inflammation, Blood Toxicity, and Hematoma

**Figure 4:**
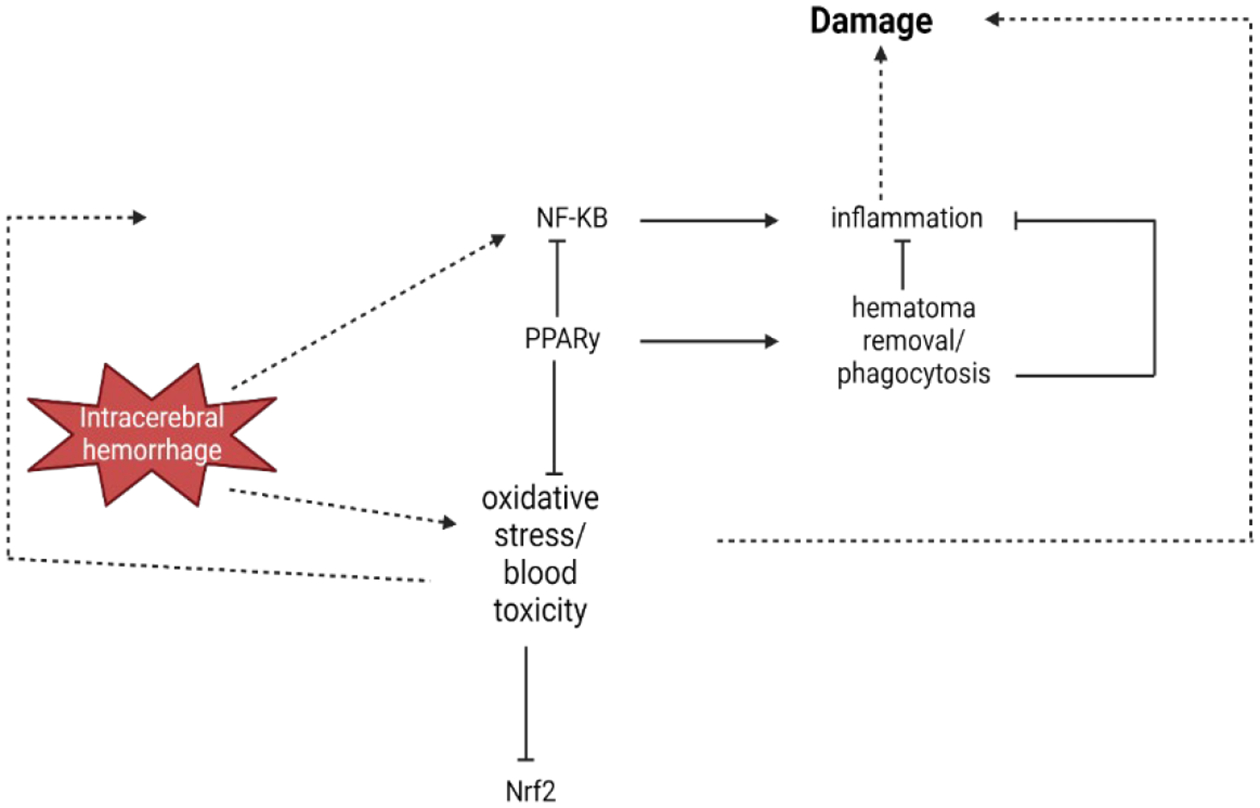
When an intracerebral hemorrhage occurs, it triggers the activation of a transcription factor called nuclear factor kappa-B (NF-κB). This activation sets off a chain reaction of inflammation and oxidative stress, ultimately causing additional damage to the brain. By inhibiting NF-κB and inducing antioxidative defence components, the transcription factor peroxisome proliferator-activated receptor-gamma (PPARγ) helps to reduce inflammation and oxidative stress, ultimately protecting the brain affected by ICH. As a biologist, it is fascinating to observe the role of the transcription factor NF-E2–related factor-2 (Nrf2) in regulating oxidative stress and blood detoxification components. Furthermore, PPARγ promotes the process of phagocytosis, which aids in the clearance of hematoma. This helps to eliminate the hematoma, which is the cause of toxicity and inflammation.

Within minutes following ICH, the brain’s resident phagocytes, microglia, which make up 10%−15% of all glial cells, are ready to go into action [111]. The ICH damage sites are infiltrated by hematogenous inflammatory cells due to the proinflammatory cytokines and chemotactic factors released by activated microglia. A brief infiltration of neutrophils (lasting 18 hours to 4 days) and a subsequent long-term presence of hematogenous macrophages (lasting 1 day to months) are the defining features of this [111]. Nuclear factor kappa-B (NF-κB) is a ubiquitous transcription factor that mainly coordinates inflammatory signaling, which incorporates several components and cell types [112]. Some of the genes that NF-κB targets include adhesion molecules (such as intercellular adhesion molecule-1), cytokines, chemokines, metalloproteinases, immune receptors, acute phase proteins, cell surface receptors, and inflammatory enzymes. Some of these genes are involved in proinflammatory signaling and NF-κB activation (Figure 4). Free radicals play a crucial role in NF-κB activation as signaling molecules. One possible explanation for how oxidative stress can worsen inflammation following ICH is this NF-κB characteristic. It has been shown in experimental experiments that NF-κB is activated in the hemisphere affected by ICH as early as 15 minutes after the start of ICH, reaches its peak between 1 and 3 days after ICH, and stays elevated for weeks. Important genes that are targeted by NF-κB, including as IL-1β, tumor necrosis factor-α, and matrix metalloproteinase-9, play a role in brain injury caused by ICH [113] (Figure 4).

### 8. Treatment Patterns of Ischemic Stroke vs Hemorrhagic Stroke

How a stroke is treated differs from one kind of stroke to another. The majority of strokes occur as a result of blocked or narrowed blood arteries, which limits blood flow to the brain. In order to treat the condition, blood-clot-busting medications are administered to the injured area. Drugs that dissolve blood clots can be administered to patients in a variety of ways, including injections, oral medication, or even straight into the brain. A carotid endarterectomy can be done to open up the carotid artery if it has become obstructed due to fat or arterial plaque. The buildup of pressure in the brain, caused by blood leaking into it from a ruptured blood vessel, is known as a hemorrhagic stroke. Treatment focuses on lowering blood pressure and regulating bleeding. Surgical repair of blood vessels may be part of emergency care. Depending on the extent of the stroke and the area of the brain that was injured, rehabilitation after a stroke may be necessary.

## 9. Similarities in the Care of Ischemic and Non-ischemic Strokes

### Emergency Care

Emergency medical treatment is crucial in both ischemic and non-ischemic strokes in order to reduce brain damage and maximize recovery time.

In the event of an ischemic stroke, the primary objective of treatment is to return blood circulation to the afflicted region of the brain in the initial hours following the start of stroke symptoms. If a patient has a blood clot obstructing one of the major cerebral arteries, a mechanical thrombectomy may be an option. The procedure entails threading a catheter into the artery so the device may dislodge the clot. A lessening of stroke-related impairment over time is possible with this treatment. The optimal time to undertake mechanical thrombectomy after a stroke symptom has begun is within six hours. In some cases (based on brain imaging test results), it can even be helpful for up to 24 hours after symptoms begin. It is more probable that the treatment will be helpful if administered promptly. Due to the serious nature of the condition, patients experiencing intracerebral hemorrhage as a result of hypertension require immediate medical attention. The doctor may suggest surgery to lower the pressure inside the skull, stop the bleeding, or both. The time it takes to do surgery following a stroke varies from 48 to 72 hours and is based on the patient’s state and the severity of the stroke.

### Diagnostic Imaging

In order to diagnose ischemic and hemorrhagic strokes, healthcare providers must have access to imaging modalities such as computed tomography (CT) or magnetic resonance imaging (MRI). This allows them to evaluate the level of damage and devise treatment plans accordingly.

When diagnosing and treating an ischemic stroke, doctors frequently use imaging modalities such as computed tomography (CT) and magnetic resonance imaging (MRI). The perfusion status of brain tissue can be evaluated using these modalities, and they also confirm the diagnosis of ischemic stroke. Ischemic cores are irreparably destroyed, while penumbra tissues may be recoverable; these markers help to differentiate between the two. If a patient with an ischemic stroke has a detectable ischemic lesion on DWI and no hyperintense signal in the same area on FLAIR, and the time of symptom onset is known to be within 4.5 hours before imaging, then the patient is likely to have symptoms within that time frame, according to MRI studies in individuals with this condition [114]. Differentiating between core and penumbra can also be achieved by CTP imaging [115]. When the time of ischemic stroke onset is uncertain or outside the 4.5–6 h time window, these two modalities have been employed in an extended time window to select patients who are likely to benefit from multiple reperfusion methods, including IVT and EVT. Additional tools that can help determine the cause of an ischemic stroke include magnetic resonance angiography (MR angiography) and computed tomography (CTA) [116]. use cutting-edge imaging techniques, including computed tomography (CT) scans or positron emission tomography (MRI), to detect hemorrhagic stroke. brain scan using computed tomography. If you suspect brain bleeding, a computed tomography (CT) scan is your gold standard for diagnosis. These scans can also show you how the bleeding is progressing in the first six hours after it starts. When it comes to diseased brain images, particularly longitudinal images and lesion foci, cranial magnetic resonance imaging (MRI) can be more precise.

### Surgical Interventions

Craniotomy and other surgical procedures may be necessary to drain blood from the brain after a hemorrhagic stroke or to fix vascular anomalies such as AVMs or aneurysms. While surgical procedures are less prevalent in cases of ischemic stroke, mechanical thrombectomy—a procedure to remove blood clots from blocked arteries—may be necessary in some cases.

In order to prevent further bleeding after a hemorrhagic stroke, a surgeon will insert a small clamp into the opening of the aneurysm. This clamp has the dual purpose of preventing the aneurysm from either bursting or further bleeding once it has already occurred. If your AVM is small and situated in an accessible part of your brain, your surgeon may be able to remove it. This reduces the likelihood of hemorrhagic stroke and removes the possibility of rupture. A physician will insert a catheter into your carotid arteries via a groin artery during an angioplasty, a surgical procedure that involves repairing or unblocking an artery. The next step is to blow up a balloon in order to widen the constricted artery. After the artery is opened, a stent can be placed to provide support. After a stroke caused by ischemia, if a blood clot is obstructing a major artery in the brain, a mechanical thrombectomy may be an option. It entails threading a device that can dislodge the clot into the artery through a catheter. Reducing stroke-related impairment over the long run is possible with this surgery.

## 10. Differences in the Care of Ischemic and Non-ischemic Strokes

### Treatment Goals

A primary goal in treating ischemic stroke is to remove or dissolve blood clots so that blood may once again flow to the damaged brain area. Hemorrhagic stroke treatment, on the other hand, focuses on addressing underlying vascular problems, controlling bleeding, and reducing brain pressure.

The majority of strokes are ischemic strokes, which occur when blood arteries supplying the brain are either completely or partially closed off due to clots or atherosclerosis. Treatment for ischemic strokes typically involves reestablishing blood flow to the afflicted area of the brain by means of clot-busting medications. The dissolving of blood clots can be accomplished by administering injections or oral drugs to patients. Potentially effective treatments include drugs administered intravenously to the brain. A procedure known as carotid endarterectomy can be performed to open up the carotid artery and allow blood to flow again to the brain in cases where it has been clogged due to arterial plaque or fat accumulation within the artery walls. The technique involves the surgeon physically removing the plaque after making an incision in the artery. Treating a hemorrhagic stroke entails lowering brain pressure and stopping any bleeding that may have occurred. The extent of the bleeding after a hemorrhagic stroke determines whether a craniotomy, or skull-based surgery, is necessary to drain the blood and alleviate pressure on the brain. Hemorrhagic strokes can cause damage to blood vessels, which can be fixed through surgery.

### Medication

While thrombolytic therapy is commonly used to treat ischemic stroke by dissolving blood clots, it is not normally used to treat hemorrhagic stroke because of the danger of worsening bleeding.

When treating an ischemic stroke, the sole medication that has been licensed for systemic administration is intravenous plasminogen activator (IVA) with alteplase [117]. This medication should be given to the patient within four and a half hours of the start of symptoms. If a large vessel occlusion (LVO) is detected, intravenous thrombolysis (IVT) can be performed either independently or in conjunction with extravascular thrombolysis (EVT) and mechanical thrombectomy (MT). It is advised that patients with LVO undergo MT within 6 hours of the start of symptoms, either alone or in conjunction with IVT within 4.5 hours of the start of symptoms, or between 4.5 and 6 hours of the beginning of symptoms. The dissolving of blood clots can be accomplished by administering injections or oral drugs to patients. Potentially effective treatments include drugs administered intravenously to the brain. Thrombolytic treatment entails intravenous (IV) administration of alteplase (or tPA, short for “tissue plasminogen activator”) or a comparably named Tenecteplase. A clot that is preventing blood from reaching the brain can be broken up by this. Anticoagulation is rarely used to treat acute hemorrhagic stroke due to the danger of severe bleeding.

## 11. Conclusion

Stroke is a leading cause of death and disability worldwide. Ischemic and hemorrhagic strokes are now better understood and treated thanks to new diagnostic methods, improved categorization systems, and other technological advancements. The two main causes of stroke are occlusion of blood vessels (ischemic stroke) and rupture of brain vessels (hemorrhagic stroke). The key to effective management and prevention is a thorough understanding of these processes and risk factors. Stroke treatment plans differ in approach based on stroke severity; nevertheless, reversing blood flow, lowering brain pressure, and treating underlying vascular problems are common goals. While surgical procedures, mechanical thrombectomy, and thrombolytic therapy are part of acute management, risk factor management and healthy lifestyle promotion are at the heart of long-term preventative efforts. Reduce the worldwide burden of stroke and improve outcomes through more research into stroke pathogenesis, neuroprotective techniques, and personalized treatment options.

## Key points, Outstanding questions and Challenges

- Blood cytotoxicity, oxidative stress, and inflammation are key factors contributing to secondary injury in both stroke types.
- Mechanical thrombectomy and thrombolytic therapy are essential in the treatment of ischemic stroke, but they must be administered at the exact right time and to the right patients.
- Though mechanical thrombectomy has changed the game for treating ischemic stroke, there are still big obstacles in terms of availability and when the procedure may be done.
- There is hope for improving recovery from all forms of stroke through neuroprotective therapies that target inflammation, oxidative stress, and excitotoxicity.
- How could the strategies/methods in personalized medicine be seamlessly incorporated into standard stroke care to get the best possible treatment results?
- What role may new discoveries in biomarkers and neuroimaging play in enhancing the ability to detect and predict the impact of strokes in their early stages?

## References

[1] Sacco RL, Kasner SE, Broderick JP, An updated definition of stroke for the 21st century: a statement for healthcare professionals from the American heart association/American stroke association. Stroke 44 (2013): 2064–2089. 23652265 10.1161/STR.0b013e318296aeca PMC11078537

[2] Mandal A Cerebral Infarction. News (2019).

[3] Hemorrhagic Stroke. Www.Stroke.Org, www.stroke.org/en/about-stroke/types-of-stroke/hemorrhagic-strokes-bleeds. Accessed 27 Mar. 2024.

[4] Experts PNI. What is the Difference between Hemorrhagic and Ischemic Stroke?” Pacific Neuroscience Institute, 3 Mar. 2023, www.pacificneuroscienceinstitute.org/blog/stroke/what-is-the-difference-between-hemorrhagic-and-ischemic-stroke/

[5] O’Donnell M, Xavier D, Diener C, Rationale and design of INTERSTROKE: a global case-control study of risk factors for stroke. Neuroepidemiology 35 (2010): 36–44. 20389123 10.1159/000306058

[6] O’Donnell MJ, Denis X, Liu L, Risk factors for ischaemic and intracerebral haemorrhagic stroke in 22 countries (the INTERSTROKE study): a case-control study. The Lancet 376 (2010): 112–123. 10.1016/S0140-6736(10)60834-3 20561675

[7] Sarfo FS, Ovbiagele B, Gebregziabher M, Stroke Among Young West Africans. Stroke 49 (2018): 1116–1122. 29618553 10.1161/STROKEAHA.118.020783 PMC5916042

[8] Chen PH, Gao S, Wang YJ, Classifying Ischemic Stroke, from TOAST to CISS. CNS neuroscience and therapeutics 18 (2012): 452–6. 22268862 10.1111/j.1755-5949.2011.00292.x PMC6493455

[9] Radu RA, Terecoasă EO, Băjenaru OA, Etiologic classification of ischemic stroke: Where do we stand? Clinical Neurology and Neurosurgery 159 (2017): 93–106. 28609703 10.1016/j.clineuro.2017.05.019

[10] Donkor Eric S Stroke in the 21st Century: A Snapshot of the Burden, Epidemiology, and Quality of Life. Stroke research and treatment (2018): 3238165. 30598741 10.1155/2018/3238165 PMC6288566

[11] Unnithan AKA, Das JM, Mehta P. Hemorrhagic Stroke. [Updated 2023 May 8]. In: StatPearls [Internet]. Treasure Island (FL): StatPearls Publishing (2024). Available from: https://www.ncbi.nlm.nih.gov/books/NBK559173/32644599

[12] Ziu E, Suheb MZK, Mesfin FB, Subarachnoid Hemorrhage. StatPearls, StatPearls Publishing (2023). 28722987

[13] Mira K, Luft A. Global Burden of Stroke. Seminars in neurology 38 (2018): 208–211. 29791947 10.1055/s-0038-1649503

[14] Tsao CW, Aday AW, Almarzooq ZI, Heart disease and stroke statistics—2022 update: a report from the American Heart Association, Circulation 145 (2022): e153–e639. 35078371 10.1161/CIR.0000000000001052

[15] Facts Stroke. Centers for Disease Control and Prevention, Centers for Disease Control and Prevention (2023).

[16] www.uptodate.com/contents/transient-ischemic-attack-beyond-the-basics/print

[17] Ovbiagele B, Nguyen-Huynh MN. Stroke epidemiology: advancing our understanding of disease mechanism and therapy.” Neurotherapeutics 8 (2011): 319–329. 21691873 10.1007/s13311-011-0053-1 PMC3250269

[18] Kokubo Y Epidemiology of transient ischemic attack.” Frontiers of neurology and neuroscience 33 (2014): 69–81. 24157557 10.1159/000351892

[19] Feigin VL, Roth GA, Naghavi M, Global burden of stroke and risk factors in 188 countries, during 1990–2013: a systematic analysis for the Global Burden of Disease Study 2013, Lancet Neurol 15 (2016): 913–924. 10.1016/S1474-4422(16)30073-4 27291521

[20] O’Donnell MJ, Chin SL, Rangarajan S, Global and regional effects of potentially modifiable risk factors associated with acute stroke in 32 countries (INTERSTROKE): a case-control study. Lancet (London, England) 388 (2016): 761–75. 27431356 10.1016/S0140-6736(16)30506-2

[21] Kleindorfer DO, Towfighi A, Chaturvedi AS, 2021 guideline for the prevention of stroke in patients with stroke and transient ischemic attack: a guideline from the American Heart Association/American Stroke Association, Stroke 52 (2021): e364–e467. 34024117 10.1161/STR.0000000000000375

[22] Capirossi C, Laiso A, Renieri A, Epidemiology, organization, diagnosis and treatment of acute ischemic stroke.” European Journal of Radiology Open 11 (2023): 100527. 37860148 10.1016/j.ejro.2023.100527 PMC10582298

[23] Muntner P, Carey RM, Gidding S, Potential US population impact of the 2017 ACC/AHA high blood pressure guideline. Circulation 137 (2018): 109–118. 29133599 10.1161/CIRCULATIONAHA.117.032582 PMC5873602

[24] Chobanian AV, Bakris GL, Black HR, The seventh report of the joint national committee on prevention, detection, evaluation, and treatment of high blood pressure: the JNC 7 report. The Journal of the American Medical Association 289 (2003): 2560–2572. 12748199 10.1001/jama.289.19.2560

[25] Home. Framingham Heart Study, 27 Mar. 2024, www.framinghamheartstudy.org/

[26] Gaciong Z, Siński M, Lewandowski J, Blood pressure control and primary prevention of stroke: summary of the recent clinical trial data and meta-analyses. Current hypertension Reports 15 (2013): 559–74. 24158454 10.1007/s11906-013-0401-0 PMC3838588

[27] PSC. Clinical Trial Service Unit & Epidemiological Studies Unit (CTSU) www.ctsu.ox.ac.uk/research/psc. Accessed 29 Mar. 2024.

[28] Dubow J, Fink ME. Impact of hypertension on stroke.” Current Atherosclerosis Reports 13 (2011): 298–305. 21626308 10.1007/s11883-011-0187-y

[29] Lewington S, Clarke R, Qizilbash N, Age-specific relevance of usual blood pressure to vascular mortality: a meta-analysis of individual data for one million adults in 61 prospective studies. Lancet 360 (2002): 1903–1913. 12493255 10.1016/s0140-6736(02)11911-8

[30] IDF Diabetes Atlas. IDF Diabetes Atlas, diabetesatlas.org (2024).

[31] Collaboration TERF. Diabetes mellitus, fasting blood glucose concentration, and risk of vascular disease: a collaborative meta-analysis of 102 prospective studies. Lancet 375 (2010): 2215–2222. 20609967 10.1016/S0140-6736(10)60484-9 PMC2904878

[32] Chen R, Ovbiagele B, Feng W. Diabetes and Stroke: Epidemiology, Pathophysiology, Pharmaceuticals and Outcomes. The American journal of the medical sciences 351 (2016): 380–6. 27079344 10.1016/j.amjms.2016.01.011 PMC5298897

[33] Khoury JC, Kleindorfer D, Alwell K, Diabetes mellitus: a risk factor for ischemic stroke in a large biracial population. Stroke 44 (2013): 1500–1504. 23619130 10.1161/STROKEAHA.113.001318 PMC3746032

[34] Roy-O’Reilly M, McCullough LD. Age and Sex Are Critical Factors in Ischemic Stroke Pathology. Endocrinology 159 (2018): 3120–3131. 30010821 10.1210/en.2018-00465 PMC6963709

[35] Bushnell C, McCullough LD, Awad IA, Guidelines for the prevention of stroke in women: a statement for healthcare professionals from the American Heart Association/American Stroke Association. Stroke 45 (2014): 1545–88. 24503673 10.1161/01.str.0000442009.06663.48 PMC10152977

[36] George MG, Tong X, Kuklina EV, Trends in stroke hospitalizations and associated risk factors among children and young adults, 1995–2008. Annals of Neurology 70 (2011): 713–21. 10.1002/ana.22539 21898534

[37] Moosa A, Osama D, Alnidawi F, Risk Factors, Incidence, and Outcome of Stroke: A Retrospective Cross-Sectional Hospital-Based Study Comparing Young Adults and Elderly. Cureus 15 (2023): e40614. 37476123 10.7759/cureus.40614 PMC10354461

[38] Saposnik G, Cote R, Phillips S, Stroke outcome in those over 80: a multicenter cohort study across Canada. Stroke 39 (2008): 2310–7. 18556583 10.1161/STROKEAHA.107.511402

[39] Benjamin EJ, Virani SS, Callaway CW, American Heart Association Council on prevention, statistics, stroke statistics, heart disease and stroke statistics – 2018 update. Circulation 137 (2018): e67–e492. 29386200 10.1161/CIR.0000000000000558

[40] Koton S, Schneider AL, Rosamond WD, Stroke incidence and mortality trends in US communities, 1987–2011. JAMA 312 (2014): 259–268. 10.1001/jama.2014.7692 25027141

[41] Madsen TE, Khoury J, Alwell K, Sex-specific stroke incidence over time in the Greater Cincinnati/Northern Kentucky Stroke Study. Neurology 89 (2017): 990–996. 28794254 10.1212/WNL.0000000000004325 PMC5589794

[42] Bushnell CD, Chaturvedi S, Gage KR, Sex differences in stroke: Challenges and opportunities. Journal of cerebral blood flow and metabolism 38 (2018): 2179–2191. 30114967 10.1177/0271678X18793324 PMC6282222

[43] Iadecola C, Gorelick PB: Hypertension, Angiotensin, and Stroke: Beyond Blood Pressure. Stroke 35 (2004): 348–350. 14757875 10.1161/01.STR.0000115162.16321.AA

[44] Johansson BB. Hypertension mechanisms causing stroke. Clinical and experimental pharmacology & physiology 26 (1999): 563–5. 10405790 10.1046/j.1440-1681.1999.03081.x

[45] Mosenzon O, Cheng AYY, Rabinstein AA, Diabetes and Stroke: What Are the Connections? Journal of stroke 25 (2023): 26–38. 36592968 10.5853/jos.2022.02306 PMC9911852

[46] Virani SS, Alonso A, Benjamin EJ, Heart Disease and Stroke Statistics—2020 Update: A Report From the American Heart Association. Circulation 141 (2020). 10.1161/CIR.0000000000000757 31992061

[47] Balami JS, Buchan AM. Complications of intracerebral hemorrhage. The Lancet Neurology 11 (2012): 101–118. 22172625 10.1016/S1474-4422(11)70264-2

[48] La Pira B, Singh TD, Rabinstein AA, Time Trends in Outcomes After Aneurysmal Subarachnoid Hemorrhage Over the Past 30 Years. Mayo Clinic Proceedings 93 (2018): 1786–1793. 30522593 10.1016/j.mayocp.2018.06.027

[49] Noothi SK, Ahmed MR, Agrawal DK. Residual risks and evolving atherosclerotic plaques. Mol Cell Biochem 478 (2023): 2629–2643. 36897542 10.1007/s11010-023-04689-0 PMC10627922

[50] Lopes LA, Agrawal DK. Thromboembolism in the Complications of Long COVID-19. Cardiol Cardiovasc Med 7 (2023): 123–128. 37389402 10.26502/fccm.92920317 PMC10310316

[51] Lopes LA, Agrawal DK. Post-Operative Atrial Fibrillation: Current Treatments and Etiologies for a Persistent Surgical Complication. J Surg Res (Houst) 5 (2022): 159–172. 35445200 10.26502/jsr.10020209 PMC9017863

[52] Chaudhary F, Ahmed Z, Agrawal DK: Critical Assessment of the Neurological Complications during High-Risk Anesthesia Procedures. J Surg Res (Houst). 2024;7(2):250–266. 38947250 PMC11213287

[53] Dhume AS, Agrawal DK. Inability of vascular smooth muscle cells to proceed beyond S phase of cell cycle, and increased apoptosis in symptomatic carotid artery disease. J Vasc Surg 38 (2003): 155–61. 12844105 10.1016/s0741-5214(02)75463-3

[54] Dhume AS, Soundararajan K, Hunter WJ 3rd, Comparison of vascular smooth muscle cell apoptosis and fibrous cap morphology in symptomatic and asymptomatic carotid artery disease. Ann Vasc Surg 17 (2003): 1–8. 12522697 10.1007/s10016-001-0331-1

[55] Rao VH, Kansal V, Stoupa S, MMP-1 and MMP-9 regulate epidermal growth factor-dependent collagen loss in human carotid plaque smooth muscle cells. Physiol Rep 10 (2014): e00224. 10.1002/phy2.224 PMC3966234 24744893

[56] Trinh J, Shin J, Rai V, Therapeutic Potential of Targeting p27^kip1^ in Plaque Vulnerability. Arch Intern Med Res 7 (2024): 73–79. 38737892 10.26502/aimr.0167 PMC11087066

[57] Velpuri P, Rai V, Agrawal DK. Role of sirtuins in attenuating plaque vulnerability in atherosclerosis. Mol Cell Biochem 479 (2024): 51–62. 36952068 10.1007/s11010-023-04714-2 PMC10034899

[58] Khwaja B, Thankam FG, Agrawal DK. Mitochondrial DAMPs and altered mitochondrial dynamics in OxLDL burden in atherosclerosis. Mol Cell Biochem 476 (2021): 1915–1928. 33492610 10.1007/s11010-021-04061-0

[59] Patel P, Rai V, Agrawal DK. Role of oncostatin-M in ECM remodeling and plaque vulnerability. Mol Cell Biochem 478 (2023): 2451–2460. 36856919 10.1007/s11010-023-04673-8 PMC10579161

[60] Doyle KP, Simon RP, Stenzel-Poore MP. Mechanisms of ischemic brain damage. Neuropharmacology 55 (2008): 310–318. 18308346 10.1016/j.neuropharm.2008.01.005 PMC2603601

[61] Unnithan AKA, Das JM, Mehta P. Hemorrhagic Stroke. StatPearls - NCBI Bookshelf (2023). 32644599

[62] Chamorro N, Dirnagl U, Urra X, Neuroprotection in acute stroke: targeting excitotoxicity, oxidative and nitrosative stress, and inflammation. The Lancet Neurology 15 (2016): 869–881. 27180033 10.1016/S1474-4422(16)00114-9

[63] Shaheryar ZA, Khan MA, Adnan CS, Neuroinflammatory Triangle Presenting Novel Pharmacological Targets for Ischemic Brain Injury. Frontiers in Immunology 12 (2012). 10.3389/fimmu.2021.748663 PMC8529160 34691061

[64] Lian L, Zhang Y, Liu L, Neuroinflammation in Ischemic Stroke: Focus on MicroRNA-mediated Polarization of Microglia. Frontiers in Molecular Neuroscience 13 (2021). 10.3389/fnmol.2020.612439 PMC7817943 33488360

[65] Xue Y, Nie D, Wang LJ, Microglial Polarization: Novel Therapeutic Strategy against Ischemic Stroke. Aging and Disease 12 (2021): 466. 33815877 10.14336/AD.2020.0701 PMC7990355

[66] Candelario-Jalil E, Dijkhuizen RM, Magnus T. Neuroinflammation, Stroke, Blood-Brain Barrier Dysfunction, and Imaging Modalities. Stroke 53 (2022): 1473–1486. 35387495 10.1161/STROKEAHA.122.036946 PMC9038693

[67] Jiang Y, Liu Z, Liao Y, Ischemic stroke: From pathological mechanisms to neuroprotective strategies. Frontiers in Neurology 13 (2022). 10.3389/fneur.2022.1013083 PMC9681807 36438975

[68] Godino MDC, Romera VG, Sánchez-Tomero JA, Amelioration of ischemic brain damage by peritoneal dialysis. Journal of Clinical Investigation 123 (2013): 4359–4363. 23999426 10.1172/JCI67284 PMC3784528

[69] Zaghmi A, Dopico-López A, Pérez-Mato M, Sustained blood glutamate scavenging enhances protection in ischemic stroke. Communications Biology 3 (2020). 10.1038/s42003-020-01406-1 PMC7713697 33273696

[70] da Silva‐Candal A, Pérez‐Díaz A, Santamaría M, Clinical validation of blood/brain glutamate grabbing in acute ischemic stroke. Annals of Neurology 84 (2018): 260–273. 30014516 10.1002/ana.25286

[71] Sonnewald U, Qu H, Aschner M. Pharmacology and Toxicology of Astrocyte-Neuron Glutamate Transport and Cycling. Journal of Pharmacology and Experimental Therapeutics 301 (2002): 1–6. 11907150 10.1124/jpet.301.1.1

[72] Nagy Z, Nardai S. Cerebral ischemia/repefusion injury: From bench space to bedside. Brain Research Bulletin 134 (2017): 30–37. 28625785 10.1016/j.brainresbull.2017.06.011

[73] Rothstein JD, Patel S, Regan MR, β-Lactam antibiotics offer neuroprotection by increasing glutamate transporter expression. Nature 433 (2005): 73–77. 15635412 10.1038/nature03180

[74] 10.1038/nature03180 Chouchani ET, Pell VR, Gaude E, Ischaemic accumulation of succinate controls reperfusion injury through mitochondrial ROS. Nature 515 (2014): 431–435. PMC4255242 25383517

[75] Pawluk H, Kołodziejska R, Grześk G, Increased Oxidative Stress Markers in Acute Ischemic Stroke Patients Treated with Thrombolytics. International Journal of Molecular Sciences 23 (2022): 15625. 36555265 10.3390/ijms232415625 PMC9779811

[76] Chavda V, Chaurasia B, Garg K, Molecular mechanisms of oxidative stress in stroke and cancer. Brain Disorders 5 (2022): 100029.

[77] Cheng Z, Wang L, Qu M, Mesenchymal stem cells attenuate blood-brain barrier leakage after cerebral ischemia in mice. Journal of Neuroinflammation 15 (2018). 10.1186/s12974-018-1153-1 PMC5932816 29724240

[78] Yu H, Chen X, Guo X, The clinical value of serum xanthine oxidase levels in patients with acute ischemic stroke. Redox Biology 60 (2023): 102623. 36739755 10.1016/j.redox.2023.102623 PMC9932569

[79] Burrage EN, Coblentz T, Prabhu SS, Xanthine oxidase mediates chronic stress-induced cerebrovascular dysfunction and cognitive impairment. Journal of Cerebral Blood Flow & Metabolism 43 (2023): 905–920. 10.1177/0271678X231152551 PMC10196752 36655326

[80] Vermot A, Petit-Härtlein I, Smith SME, NADPH Oxidases (NOX): An Overview from Discovery, Molecular Mechanisms to Physiology and Pathology. Antioxidants 10 (2021): 890. 34205998 10.3390/antiox10060890 PMC8228183

[81] Galluzzi L, Kepp O, Kroemer G. Mitochondria: master regulators of danger signalling. Nature Reviews Molecular Cell Biology 13 (2012): 780–788. 23175281 10.1038/nrm3479

[82] Sims NR, Muyderman H. Mitochondria, oxidative metabolism and cell death in stroke. Biochimica Et Biophysica Acta (BBA) - Molecular Basis of Disease 1802 (2010): 80–91. 10.1016/j.bbadis.2009.09.003 19751827

[83] Narendra DP, Jin SM, Tanaka A, PINK1 Is Selectively Stabilized on Impaired Mitochondria to Activate Parkin. PLoS Biology 8 (2010): e1000298. 20126261 10.1371/journal.pbio.1000298 PMC2811155

[84] Tuo Q, Zhang S, Lei P. Mechanisms of neuronal cell death in ischemic stroke and their therapeutic implications. Medicinal Research Reviews 42 (2021): 259–305. 33957000 10.1002/med.21817

[85] Jiang X, Andjelkovic AV, Zhu L, Blood-brain barrier dysfunction and recovery after ischemic stroke. Progress in Neurobiology 163–164 (2018): 144–171. 10.1016/j.pneurobio.2017.10.001 PMC5886838 28987927

[86] Xu W, Jin W, Zhang X, Remote Limb Preconditioning Generates a Neuroprotective Effect by Modulating the Extrinsic Apoptotic Pathway and TRAIL-Receptors Expression. Cellular and Molecular Neurobiology 37 (2016): 169–182. 26971954 10.1007/s10571-016-0360-5 PMC11482232

[87] Salaudeen MA, Bello N, Danraka RN, Understanding the Pathophysiology of Ischemic Stroke: The Basis of Current Therapies and Opportunity for New Ones. Biomolecules 14 (2024): 305. 38540725 10.3390/biom14030305 PMC10968326

[88] Qureshi AI, Mendelow AD, Hanley DF. Intracerebral hemorrhage. The Lancet 373 (2009): 1632–1644. 10.1016/S0140-6736(09)60371-8 PMC3138486 19427958

[89] Wagner KR, Sharp FR, Ardizzone TD, Heme and Iron Metabolism: Role in Cerebral Hemorrhage. Journal of Cerebral Blood Flow & Metabolism 23 (2003): 629–652. 12796711 10.1097/01.WCB.0000073905.87928.6D

[90] Ardizzone TD, Lu A, Wagner KR, Glutamate Receptor Blockade Attenuates Glucose Hypermetabolism in Perihematomal Brain After Experimental Intracerebral Hemorrhage in Rat. Stroke 35 (2004): 2587–2591. 15375303 10.1161/01.STR.0000143451.14228.ff

[91] Qureshi AI, Ali Z, Suri MFK, Extracellular glutamate and other amino acids in experimental intracerebral hemorrhage: An in vivo microdialysis study. Critical Care Medicine 31 (2003): 1482–1489. 12771622 10.1097/01.CCM.0000063047.63862.99

[92] Mun-Bryce S, Wilkerson AC, Papuashvili N, Recurring episodes of spreading depression are spontaneously elicited by an intracerebral hemorrhage in the swine. Brain Research 888 (2001): 248–255. 11150481 10.1016/s0006-8993(00)03068-7

[93] Wang J, Doré S. Inflammation after Intracerebral Hemorrhage. Journal of Cerebral Blood Flow & Metabolism 27 (2006): 894–908. 10.1038/sj.jcbfm.9600403 17033693

[94] Huang FP, Xi G, Keep RF, Brain edema after experimental intracerebral hemorrhage: role of hemoglobin degradation products. Journal of Neurosurgery 96 (2002): 287–293. 11838803 10.3171/jns.2002.96.2.0287

[95] Zhao X, Sun G, Zhang J, Hematoma resolution as a target for intracerebral hemorrhage treatment: Role for peroxisome proliferator‐activated receptor γ in microglia/macrophages. Annals of Neurology 61 (2007): 352–362. 17457822 10.1002/ana.21097

[96] Chen S, Zeng L, Hu Z. Progressing haemorrhagic stroke: categories, causes, mechanisms and managements. Journal of Neurology 261 (2014): 2061–2078. 24595959 10.1007/s00415-014-7291-1 PMC4221651

[97] An SJ, Kim TJ, Yoon BW. Epidemiology, Risk Factors, and Clinical Features of Intracerebral Hemorrhage: An Update. Journal of Stroke 19 (2017): 3–10. 28178408 10.5853/jos.2016.00864 PMC5307940

[98] Magid-Bernstein J, Girard R, Polster S, Cerebral Hemorrhage: Pathophysiology, Treatment, and Future Directions. Circulation Research 130 (2022): 1204–1229. 35420918 10.1161/CIRCRESAHA.121.319949 PMC10032582

[99] Aronowski J, Zhao X, Molecular Pathophysiology of Cerebral Hemorrhage. Stroke 42 (2011): 1781–1786. 21527759 10.1161/STROKEAHA.110.596718 PMC3123894

[100] Matsuyama T, Okuchi K, Seki T, Perimesencephalic Nonaneurysmal Subarachnoid Hemorrhage Caused by Physical Exertion. Neurologia Medico-Chirurgica 46 (2006): 277–282. 16794347 10.2176/nmc.46.277

[101] Coelho LGBSA, Costa JMD, Silva EIPA. Non-aneurysmal spontaneous subarachnoid hemorrhage: perimesencephalic versus non-perimesencephalic. Revista Brasileira De Terapia Intensiva 28 (2016). 10.5935/0103-507X.20160028 PMC4943051 27410409

[102] Kushner MJ, Bressman SB. The clinical manifestations of pontine hemorrhage. Neurology 35 (1985): 637–637. 3990963 10.1212/wnl.35.5.637

[103] Kitagawa K Blood pressure management for secondary stroke prevention. Hypertension Research 45 (2022): 936–943. 35437312 10.1038/s41440-022-00908-1

[104] Delgado Almandoz JE, Romero JM. Advanced CT Imaging in the Evaluation of Hemorrhagic Stroke. Neuroimaging Clinics of North America 21 (2011): 197–213. 21640295 10.1016/j.nic.2011.01.001

[105] Schlunk F, Kuthe J, Harmel P, Volumetric accuracy of different imaging modalities in acute intracerebral hemorrhage. BMC Medical Imaging 22 (2022). 10.1186/s12880-022-00735-3 PMC8760700 35033012

[106] Takeuchi S, Wada K, Nagatani K, Decompressive hemicraniectomy for spontaneous intracerebral hemorrhage. Neurosurgical Focus 34 (2013): E5. 10.3171/2013.2.FOCUS12424 23634924

[107] Smith EE, Rosand J, Greenberg SM. Hemorrhagic Stroke. Neuroimaging Clinics of North America 15 (2005): 259–272. 16198939 10.1016/j.nic.2005.05.003

[108] Laskowitz DT, Kolls BJ. Neuroprotection in Subarachnoid Hemorrhage. Stroke 41 (2010). 10.1161/STROKEAHA.110.595090 PMC3376008 20876512

[109] Lin J, Cai C, Xie Y, Acute glycemic variability and mortality of patients with acute stroke: a meta-analysis. Diabetology & Metabolic Syndrome 14 (2022). 10.1186/s13098-022-00826-9 PMC9092773 35538585

[110] Wang J, Rogove AD, Tsirka AE, Protective role of tuftsin fragment 1‐3 in an animal model of intracerebral hemorrhage. Annals of Neurology 54 (2003): 655–664. 14595655 10.1002/ana.10750

[111] 10.1002/ana.10750 Zhao X, Zhang Y, Strong R, Distinct patterns of intracerebral hemorrhage‐induced alterations in NF‐κB subunit, iNOS, and COX‐2 expression. Journal of Neurochemistry 101 (2006): 652–663. 17250675

[112] Tang J, Liu J, Zhou C, MMP-9 Deficiency Enhances Collagenase-Induced Intracerebral Hemorrhage and Brain Injury in Mutant Mice. Journal of Cerebral Blood Flow & Metabolism 24 (2004): 1133–1145. 10.1097/01.WCB.0000135593.05952.DE 15529013

[113] Thomalla G, Cheng B, Ebinger M, DWI-FLAIR mismatch for the identification of patients with acute ischaemic stroke within 4·5 h of symptom onset (PRE-FLAIR): a multicentre observational study. The Lancet Neurology 10 (2011): 978–986. 21978972 10.1016/S1474-4422(11)70192-2

[114] Lansberg MG, Christensen S, Kemp S, Computed tomographic perfusion to Predict Response to Recanalization in ischemic stroke. Annals of Neurology 81 (2017): 849–856. 28486789 10.1002/ana.24953 PMC5521988

[115] Kilburg C, Scott McNally J, de Havenon A, Advanced imaging in acute ischemic stroke. Neurosurgical Focus 42 (2017): E10. 10.3171/2017.1.FOCUS16503 28366054

[116] Hacke W, Kaste M, Bluhmki E, Thrombolysis with alteplase 3 to 4.5 hours after acute ischemic stroke. N Engl J Med 359 (2008): 1317–29. 18815396 10.1056/NEJMoa0804656

[117] Jovin TG, Chamorro A, Cobo E, Thrombectomy within 8 hours after symptom onset in ischemic stroke. N Engl J Med 372 (2015): 2296–306. 25882510 10.1056/NEJMoa1503780

